# Independent effects of adding weight and inertia on balance during quiet standing

**DOI:** 10.1186/1475-925X-11-20

**Published:** 2012-04-16

**Authors:** Kerry Elizabeth Costello, Sara Louise Matrangola, Michael Lawrence Madigan

**Affiliations:** 1 Virginia Tech – Wake Forest School of Biomedical Engineering and Sciences, Blacksburg, VA, 24061, USA; 2 Virginia Tech – Wake Forest School of Biomedical Engineering and Sciences, Blacksburg, VA, 24061, USA; 3 Engineering Science and Mechanics (0219), Virginia Tech – Wake Forest School of Biomedical Engineering and Sciences, Blacksburg, VA, 24061, USA

**Keywords:** Balance, Center of pressure, Mass moment of inertia, Weight

## Abstract

**Background:**

Human balance during quiet standing is influenced by adding mass to the body with a backpack, with symmetrically-applied loads to the trunk, or with obesity. Adding mass to the body increases both the weight and inertia of the body, which theoretically could provide counteracting effects on body dynamics and balance. Understanding the independent effects of adding weight and inertia on balance may provide additional insight into human balance that could lead to novel advancements in balance training and rehabilitation. Therefore, the purpose of this study was to investigate the independent effects of adding weight and inertia on balance during quiet standing.

**Methods:**

Sixteen normal-weight young adult participants stood as still as possible on a custom-built backboard apparatus under four experimental conditions: baseline, added inertia only, added weight only, and added inertia and weight.

**Results:**

Adding inertia by itself had no measurable effect on center of pressure movement or backboard movement. Adding weight by itself increased center of pressure movement (indicated greater effort by the postural control system to stand as still as possible) and backboard movement (indicating a poorer ability of the body to stand as still as possible). Adding inertia and weight at the same time increased center of pressure movement but did not increase backboard movement compared to the baseline condition.

**Conclusions:**

Adding inertia and adding weight had different effects on balance. Adding inertia by itself had no effect on balance. Adding weight by itself had a negative effect on balance. When adding inertia and weight at the same time, the added inertia appeared to lessen (but did not eliminate) the negative effect of adding weight on balance. These results improve our fundamental understanding of how added mass influences human balance.

## Background

Balance control during quiet standing is influenced by changes in the mass/inertial characteristics of the body. Several studies have reported increases in center of pressure (COP) movement during load carriage with a backpack in adolescents [1-3], college students [4], and United States Army soldiers [5]. Obesity also changes the mass/inertial characteristics of the body, and studies have reported increased COP movement during quiet standing among obese compared to healthy-weight individuals [6-9] as increases in mean COP speed, mean COP position, and peak COP position with increasing body weight [10]. Backpacks and obesity not only alter the mass/inertial characteristics of the body, but they also displace the whole-body center of mass (COM) [11]. Studies have tried to separate the effects of adding mass and displacing the COM by adding mass symmetrically with respect to the mid-sagittal and frontal planes. These studies reported not only an increase in COP movement with added mass [12-14], but also a dependence upon the direction of displacement of the COM with the added mass. COP movement decreased if the COM was lowered from its natural position [13] and increased if the COM was raised from its natural position [13,14].

Adding mass to the body though a backpack, obesity, or symmetrically-applied loads has two simultaneous, and potentially counteracting, effects on body dynamics. First, adding mass increases the magnitude of the gravitational force (i.e. weight) of the body. Consider a sagittal plane inverted pendulum model of the body during quiet standing [15] with the following equation of motion:

(1) $$\ddot{\theta }(t)=\frac{WL\sin\theta (t)+M(t)}{I}$$

where *W* = weight or gravitational force of pendulum (i.e. *mg*), *L* = distance from the ankles to COM, *θ* = angle of pendulum from vertical, *t* = time, *I* = mass moment of inertia of pendulum about the ankles, and *M* = net muscle moment about the ankles. Increasing the weight of the body would seem to challenge the balance control system by increasing the angular acceleration of the body and thus requiring a larger net muscle moment and rate of change of net muscle moment at the ankles to maintain an upright posture. In addition, larger muscle forces required for larger net muscle moments are associated with increased force unsteadiness [16-18]. Increases in net muscle moment, rate of change of net muscle moment, and muscle force unsteadiness would lead to greater COP displacement, speed, and unsteadiness during quiet standing, all of which are associated with poorer balance control.

The second effect that adding mass to the body has on body dynamics is an increase in the mass moment of inertia (inertia) of the body. Again considering an inverted pendulum model of the body (Equation 1), increasing the inertia of the body (without increasing weight of the body) would decrease the angular acceleration of the body for a given angular displacement from vertical and decrease the natural frequency of the body [19], which could in effect “slow down” the system and mitigate the negative effects of control delays within the balance control system [20]. In fact, Goh et al. (1998) suggested that increases in inertia of the body with a backpack would be beneficial for balance [21]. Two common examples of increasing inertia to improve balance is the tendency to abduct the shoulders 90 ° from the anatomical position when walking across a narrow support, or for tightrope walkers to use balancing poles.

While independently manipulating the weight and inertia of the body may not seem immediately practical, understanding their independent effects on balance may lead to improved understanding of factors affecting human balance as well as changes in the mechanics of balance with added mass, such as obesity. In addition, the prospect of human travel back to the moon or to Mars may raise the importance of human balance in environments with altered gravitational fields that would lead to weight (but not inertia) differences compared to Earth. Therefore, the purpose of this study was to investigate the independent effects of adding weight and inertia on balance during quiet standing. Based upon the inverted pendulum model and the literature summarized above, it was hypothesized that 1) adding weight would impair balance, 2) adding inertia would improve balance, and 3) adding both weight and inertia would impair balance, but to a lesser extent than adding weight alone.

## Methods

Sixteen male adults participated in this study (age: 22.1 ± 1.7 year, BMI: 22.9 ± 2.0 kg/m^2^, height: 174.9 ± 5.1 cm, mass: 70.2 ± 7.7 kg). Participants were excluded if they reported any musculoskeletal injury within the past three months. This research was approved by the Virginia Tech Institutional Review Board, and written informed consent was obtained from all participants prior to participation.

Balance measurements were taken while participants attempted to stand as still as possible under four experimental conditions: baseline without added inertia or weight (B), added inertia only (I), added weight only (W), and added inertia and weight (IW). The order of presentation of the conditions was randomized across participants, and multiple quiet standing trials were performed in each condition. For the purpose of this study, “inertia” was operationally defined to be the mass moment of inertia of the body about a mediolateral axis through both ankles, and ‘weight’ was operationally defined to be the moment about a mediolateral axis through both ankles that tends to rotate the body forward (due to gravity because the COM is anterior the ankles during quiet standing).

**Figure 1:**
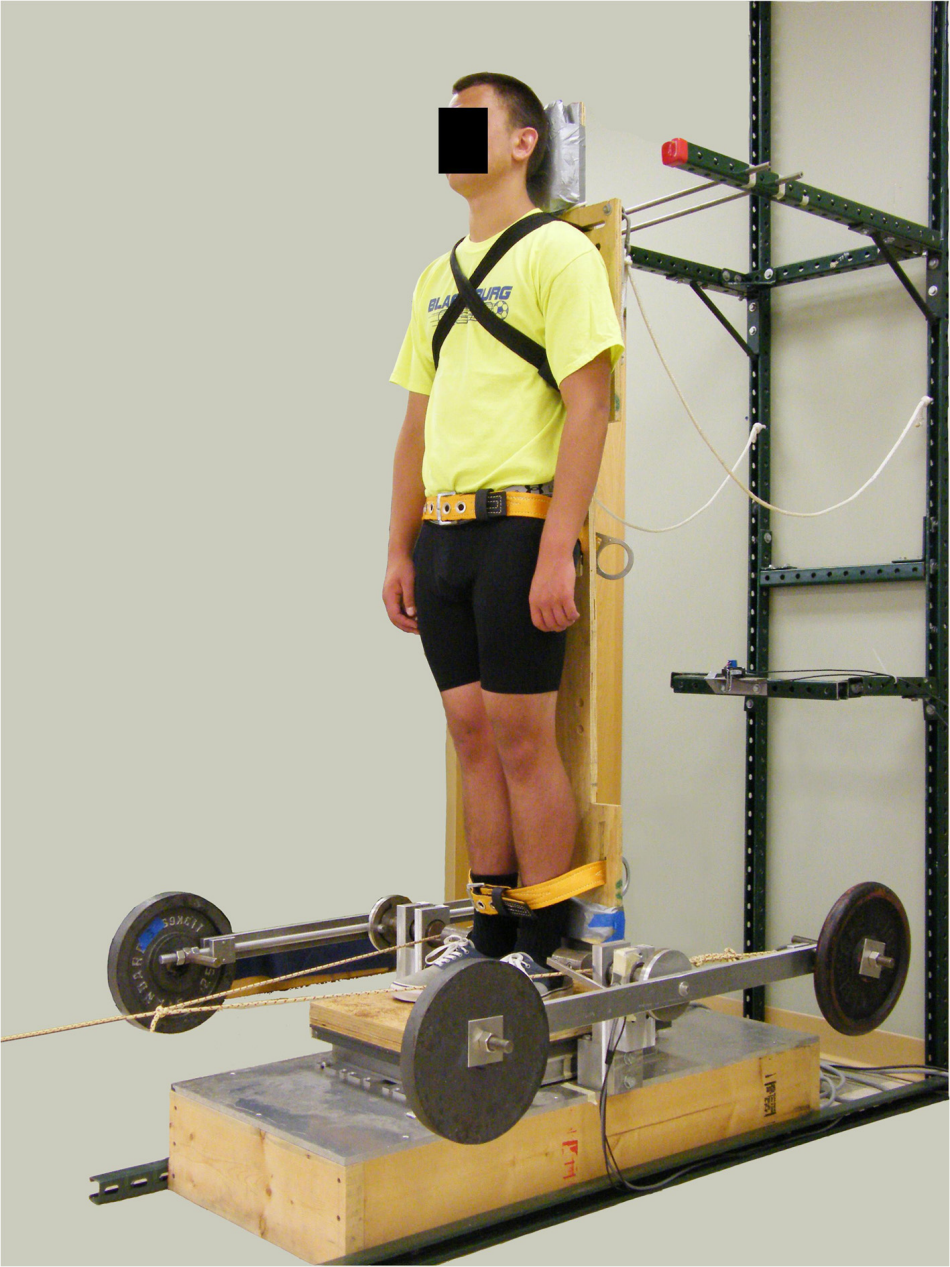
**Backboard setup showing the added inertia and weight (IW) condition.** The backboard and arms all rotate as one unit about the pin supports that were aligned with the participant’s lateral malleoli. Boards placed on top of the forceplate were used to adjust the height of the lateral malleoli in order to align them with the pin support.

A custom-built backboard apparatus was designed to allow inertia and weight to be added independently (Figure 1). The backboard was supported by two pin supports on each side of the backboard and pivoted about a mediolateral axis aligned with the lateral malleoli of participants. It thus limited movement to the sagittal plane. Wooden boards placed under the participants’ feet were used to raise or lower the feet to align their lateral malleoli with the axis of rotation of the backboard. Rigidly attached to the backboard were four “arms” (two on the participant’s left and two on the participant’s right) extending from the axis of rotation of the backboard. These arms rotated with the backboard, and were used to add inertia and weight as described below. The backboard (including arms) had a mass of 34.7 kg and a mass moment of inertia of 9.62 kg·m^2^ about the axis of rotation of the backboard during testing.

**Figure 2:**
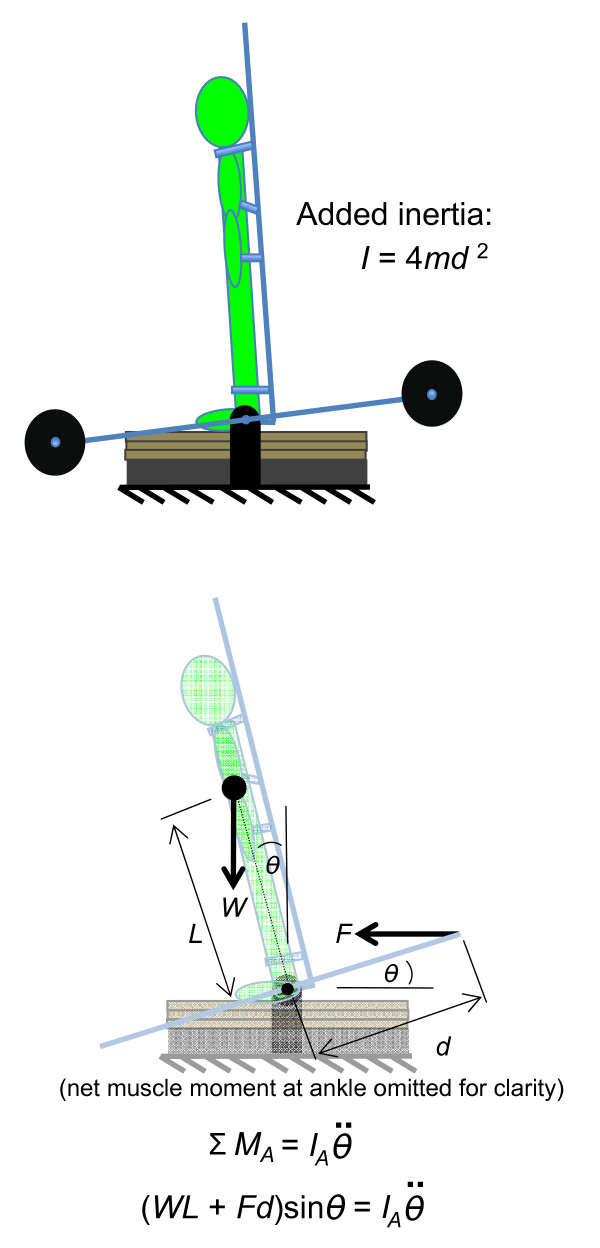
**Diagram of methods used to add inertia and weight to the backboard.** (**a**) Inertia was added by adding masses (plate weights used for resistance training depicted as black circles) symmetrically about the pin support. The added inertia from each plate weight was *md*^2^, so the added inertia of all four plate weights was 4*md*^2^. (**b**) Weight was added by applying a horizontal force (*F*) to the back end of the arms, which resulted in a moment about the pin support that tended to rotate the body forward.

To add inertia to the backboard (and thus to the backboard/body system) without adding weight, equal masses (plate weights used for resistance training) were placed symmetrically on all four of the arms attached to the backboard (Figure 2a). All four masses were positioned an equal distance from the axis of rotation, and thus did not result in a net moment about the axis of rotation (i.e. did not add weight to the body). Additionally, participants were not required to support these added masses because the backboard and arms were supported by the ground. Therefore, these masses only increased the mass moment of inertia of the backboard. Varying amounts of inertia could be added by adjusting either the amount of mass added to the arms or the distance between the masses and the axis of rotation.

**Figure 3:**
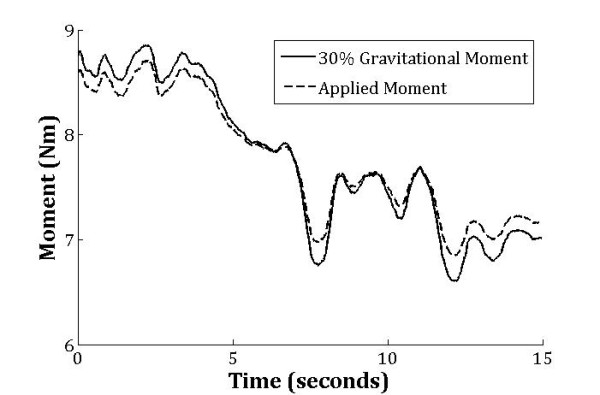
**Sample data illustrating the moment applied by surgical tubing (dashed line) compared to a 30 % increase in gravitational moment (solid line).** The root-mean-squared error between these two data series is 0.1294 Nm.

To add weight to the backboard (and thus the backboard/body system) without adding inertia, an anteriorly-directed force was applied to the ends of the two posterior backboard arms (Figure 2b). These forces caused a moment about the axis of rotation that tended to rotate the body/backboard system forward. This moment was in the same direction as the gravitational moment about the ankles, and thus acted to simulate the effects of gravity. The force was applied with stretched lightweight surgical tubing to avoid adding appreciable mass (and thus inertia) to the system. The change in length of the surgical tubing during testing was negligible, resulting in a near-constant force applied to the arms of the backboard. In order for this applied force to have the same effect on the body as gravity, it was important for the moment arm of the applied force to vary with backboard angle in the same manner that the moment arm of the gravitational force of the body applied at the COM varied with backboard angle. When the combined backboard/body system COM was directly above the axis of rotation of the backboard, there was no gravitational moment because the moment arm of the gravitational force was zero. Thus, the moment arm of the applied force also needed to be zero when the backboard was in this same angular position. This was accomplished by first fixing the angular position of the backboard such that the backboard/body system COM was above the axis of rotation, then adjusting the angle of the arms on the backboard such that the line of action of the applied force was through the middle of the axis of rotation of the backboard (which was possible due to cut-out sections of the backboard shaft). Changes in the line of action of the applied force from horizontal during quiet standing trials were negligible due to the small change in height of the tubing where connected to the posterior arms of the backboard (~ 2 cm) being orders of magnitude smaller than the 5.2 m distance from this same connection to a winch storing excess surgical tubing. As such, the moment about the ankles due to gravity and the moment about the ankles due to the applied force were both functions of the sine (*θ*) where *θ* was the angle between vertical and a line connecting the lateral malleoli with the backboard/body system COM (Figure 3). Varying amounts of weight could be added to the backboard/body system by adjusting the magnitude of the applied force. This was accomplished by tightening the surgical tubing with the winch and measuring the magnitude of the applied force using an in-line load cell (Cooper Instruments and Systems, Warrenton, VA). To simultaneously add weight and inertia to the backboard (and thus to the backboard/body system), the procedure for adding inertia and the procedure for adding weight were used simultaneously.

Several steps were completed to determine the COM position of the backboard/body system prior to performing quiet standing trials. The position of the backboard COM was determined prior to all human subject testing using the reaction board method [22]. The participant’s superior-inferior COM position was also determined using the same method. The anterior-posterior (AP) position of the backboard/body system COM for each participant was determined knowing that the mean AP position of the COM is equal to the mean AP position of the COP under the feet during quiet standing over an extended period of time [22]. Three 30-s quiet standing trials on a force plate (Bertec Corp., Columbus, OH) were collected and used to determine the mean AP position of the COP and the mean angle of the backboard with a linear potentiometer (Unimeasure, Corvallis, OR). Knowing the backboard/body system COM position in the superior-inferior direction from the reaction board method, its anterior-posterior position was determined by knowing that the COM must be positioned over the average position of the COP with the backboard at the measured average angle.

**Figure 4:**
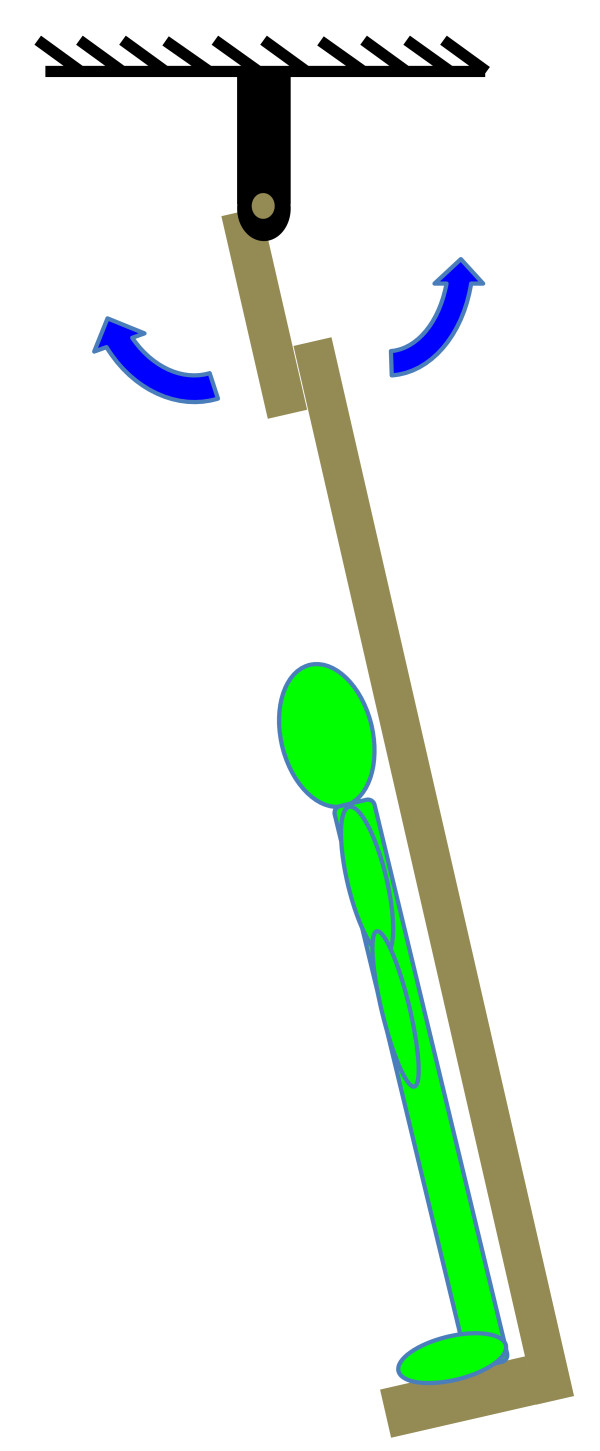
Diagram of swing used to determine the inertia of participants about a transverse axis through the lateral malleoli.

The inertia of participants was calculated using the relationship between the time period of a swinging pendulum and its inertia:

(2) $$I=\frac{T^{2}mgd_{\mathrm{COM}}}{4\pi^{2}}$$

where *I* is the inertia of the swing/body about the axis of rotation, *T* is the time period of one swing cycle, *m* is the combined mass of the swing/body, *g* is the gravitational constant (9.81 m/s^2^), and *d*_*COM*_ is distance from the axis of rotation to the combined COM of the swing/body. Equation 2 assumes no friction and a small angle approximation. Participants stood upright on a custom-built rigid wooden swing with their arms at their sides and head facing forward (Figure 4). The axis of rotation of the swing was over the head of the participants and parallel to an axis passing through both lateral malleoli. The time required for five cycles following release from an initial angle of approximately 8° from vertical was measured using a Vicon 460 motion analysis system (Vicon, Lake Forest, CA) and divided by five to obtain the time period of one swing cycle. This test was repeated five times and averaged to obtain *T* for Equation 2. Because the values for mass and distance to the COM used in Equation 1 were for the combined COM of the swing/body system, the inertia of the swing itself (77.9 kg*m^2^ about the swing’s pivot, mass of swing: 30.0 kg) was first subtracted, and then the parallel axis theorem was used to obtain the inertia of participants about a transverse axis through the lateral malleoli.

At the beginning of the experiment, height, mass, ankle height, foot length, and ankle-to-heel distance of the participant were measured. The body COM position and inertia were then determined using the methods described above. Participants then performed quiet standing trials under each of the four experimental conditions (B, I, W, and IW). A 30 % increase in inertia and/or weight was induced for the I, W, and IW experimental conditions. This 30 % increase was of the participants’ initial body inertia and/or weight (not the backboard/body system inertia and/or weight). A 30 % increase in both inertia and weight was selected because a 30 % increase in mass increased the average participant BMI from approximately 23 kg/m^2^ (near middle of normal-weight range) to approximately 30 kg/m^2^ (which is the threshold for indicating obesity). Participants were strapped to the backboard at the chest, mid-thigh, and mid-calf. They were instructed to stand as still as possible with their eyes closed, arms at their sides, and head facing forward for 30 s. After one 30-s practice trial at each experimental condition, participants completed four trials while forceplate data, backboard angle data, and load cell data were sampled at 500 Hz. All data were low-pass filtered at 10 Hz (4^th^-order Butterworth zero-phase-lag filter). Electromyography of the left tibialis anterior was monitored during testing to ensure participants did not exhibit obvious increases in lower leg co-contraction above the level observed during quiet standing without the backboard. Participants wore athletic clothing without shoes during the entire testing session.

Balance was quantified using both the COP and backboard angle. COP measures included mean COP position (the mean AP COP position in the relative to the ankle), AP range of COP position, mean AP COP speed (the mean speed of the COP trajectory), and range of AP COP speed. Backboard angle measurements included mean backboard angle (mean angle of backboard relative to vertical), range of backboard angle, mean backboard angular velocity (mean angular velocity of backboard about its pivot), and the range of backboard angular velocity. In addition, the root mean square value of the instantaneous difference between the COP and COM positions (COP-COM) [23] was calculated. A two-way repeated measures analysis of variance was conducted for each balance measurement using trial (1–4) and condition (B, I, W, IW) as independent variables. When significance was found for either independent variable or their interaction, post-hoc Tukey Honestly Significant Difference tests were performed to determine differences between conditions. A statistical significance level of *p* ≤ 0.05 was used for all analyses (JMP v7, Cary, NC, USA). Prior to statistical analysis, three balance measures (range of COP speed, range of backboard angular position, and range of backboard angular speed) were successfully corrected for a skewed distribution using a logarithmic transformation.

## Results

No main effects of trial or trial × condition interactions were statistically significant. As such, the results focus on the main effect of condition.

**Figure 5:**
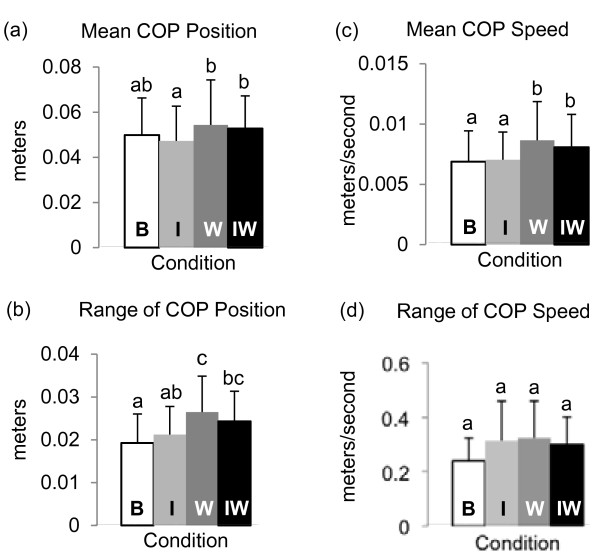
**COP measures of balance.** (**a**) Mean COP position measured from the lateral malleoli. Positive numbers indicate anterior to the lateral malleoli. (**b**) Range of COP position. (**c**) Mean COP speed. (**d**) Range of COP speed. B – baseline, I – added inertia only, W – added weight only, IW – added inertia and weight. Conditions not connected by the same letter are statistically different.

The mean COP position differed across the four conditions (*p* = 0.001), and was more anterior in both the W and IW conditions than in the I condition (Figure 5a). The range of COP position also differed across the four conditions (*p* < 0.001), and was larger in both the W and IW conditions than in the B condition (Figure 5b). The range of COP position was also larger in the W condition than in the I condition. The mean COP speed was higher in both the W and IW conditions than both the B and I conditions (*p* < 0.001; Figure 5c). Range of COP speed did not differ across conditions (*p* = 0.212; Figure 5d).

**Figure 6:**
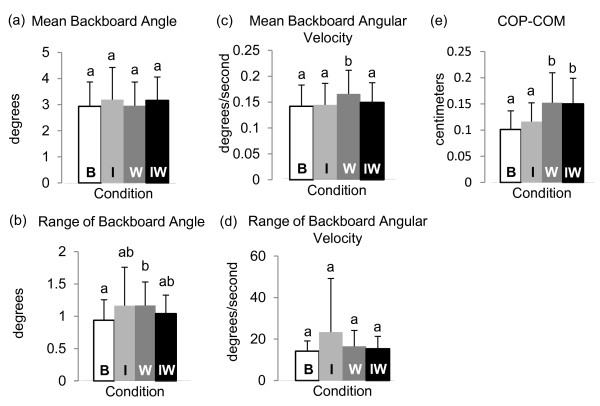
**Backboard angle measures of balance.** (**a**) Mean backboard angle (vertical is zero and increasing as backboard rotates forward). (**b**) Range of backboard angle. (**c**) Mean backboard angular velocity. (**d**) Range of backboard angular velocity. (**e**) root mean square of COP-COM (both in AP plane).

The mean backboard angle did not differ across conditions (*p* = 0.074; Figure 6a), but the range of backboard angle did differ across conditions (*p* = 0.032) with a larger range in the W condition than in the B condition (Figure 6b). Mean backboard angular velocity also differed across conditions (*p* < 0.001), and was higher in the W condition than any of the other three conditions (Figure 6c). The range of angular speed of the backboard was not different for different conditions (*p* = 0.430; Figure 6d).

COP-COM differed across conditions (*p* < 0.001) with COP-COM of both the W and IW conditions higher than both the B and I conditions (Figure 6e).

## Discussion

The purpose of this study was to investigate the independent effects of adding weight and inertia on balance during quiet standing. Adding 30 % inertia by itself had no measurable effects on balance. Adding 30 % weight by itself had a negative effect on balance, increasing six of nine measures from baseline. Simultaneously adding 30 % inertia and 30 % weight also had a negative effect of balance, but with only four of nine measures increased from baseline. As such, the added inertia appeared to mitigate to some extent the negative effects of weight on balance. Results supported the first and third hypotheses, but not the second.

Two aspects of our experimental methods warrant discussion. First, it was necessary to use a backboard to constrain movement to the sagittal plane and only allow movement at the ankles due to practical difficulties independently manipulating weight and inertia. While constraining the body above the ankles to move as one segment is consistent with the inverted pendulum model of balance [15], small but statistically significant effects of joint immobilization on quiet standing have been reported [24]. Nevertheless, these effects were consistent across all experimental conditions investigated and thus is not anticipated to largely influence the effects reported. However, it is unclear how these results would generalize to unconstrained quiet standing. Second, the term ‘weight’ typically refers to a body force due to gravity. Again due to practical difficulties independently manipulating weight and inertia, the term ‘weight’ used here was operationally defined to be the moment due to gravity about a mediolateral axis through both ankles. This operational definition did not affect the governing dynamics of the system compared to actually increasing the body force due to gravity, but may have resulted in differences in plantar pressures under the soles of the feet compared to actually increasing the body force of participants. It is unclear if this had a meaningful effect on sensory feedback from the plantar surface of the feet, but given the similarities between balance measures here and reported elsewhere, we suspect any effects to be minor.

COP measures reported here, and the direction of change of these measures with added weight and inertia, are consistent with those reported elsewhere with added body mass due to obesity. Teasdale et al. (2007) reported mean COP speed (0.82 cm/s) and anterior-posterior COP range (1.80 cm) for normal-weight young male adults with their eyes closed that were comparable to those reported here for the B condition (0.69 cm/s and 1.9 cm, respectively) [25]. Teasdale et al. (2007) also reported mean COP speed (1.16 cm/s) and COP range (2.32 cm) for obese young male adults with their eyes closed that were comparable to those reported here for the IW condition (0.81 cm/s and 2.4 cm, respectively). While comparable, the values for mean COP speed in the present study are consistently lower than those reported by Teasdale et al. (2007). This may be because the present study only calculated mean COP speed in the anterior-posterior direction whereas Teasdale et al. calculated it from total COP movement, or perhaps because Teasdale et al. tested individuals with obesity while our study tested individuals after adding weight/inertia that only simulated obesity. In addition to these quantitative comparisons, the qualitative increase in mean COP speed and lack of change in mean COP position from the B to IW condition are consistent with a previous study that investigated the effects of load carriage during standing balance. Qu and Nussbaum (2009) reported increases in mean AP COP speed and no change in COP RMS position during quiet standing after adding 10 % or 20 % additional body mass worn in a belt centered around the participant’s COM [14].

After adding 30 % inertia, participants exhibited no differences in balance measures from the baseline condition. COP speed is typically interpreted as the amount of “balancing activity” that is needed to stand as still as possible [26-28]. Therefore, the results of this study suggest that it was neither easier nor more difficult to stand as still as possible with 30 % increased inertia. Additionally, ankle torque is proportional to COP position during quiet standing [22] and thus the lack of difference in the mean COP position between the I and B conditions (as well as the lack of difference in extreme values of COP position reflected in the range of the COP position) indicated that no additional ankle torque was needed to control balance during the I condition. Therefore, it appears that adding 30 % inertia does not necessitate larger ankle torques to control larger inertial forces during quiet standing.

After adding 30 % weight, participants exhibited, on average, 37.5 % larger COP range, 25.7 % larger mean COP speed, 24.3 % larger backboard angular range, 16.9 % larger mean backboard angular speed, and 50.5 % larger COP-COM distance compared to the B condition. The increase in mean COP speed indicates greater “balancing activity” compared to the B condition. The increased COP range indicated a larger extreme value of ankle torque was used during quiet standing. Taken together, these two results suggest that standing as still as possible in the W condition was more difficult and required more effort than the B condition. Consistent with this, increased backboard angular range and speed indicated the body was moving faster and over a larger distance in the W condition than in the B condition. COP-COM gives information about the error signal responsible for controlling COM position, and is directly related to the angular acceleration of the participant [22]. Thus, a larger value for COP-COM in the W condition means that larger angular accelerations were occurring because there was a greater error in the COP position controlling the movement of the COM. This is consistent with the other results for increased weight in that it was more difficult to control movement with 30 % increased weight.

After adding 30 % weight and 30 % inertia, participants exhibited, on average, 26.2 % larger COP range, 17.3 % larger mean COP speed, and 48.6 % larger COP-COM distance compared to the B condition. Similar to the W condition, larger mean COP speed and COP range suggested more “balancing activity” and larger extreme values of ankle torque were used during quiet standing in the IW condition compared to the B condition. Similarly, the larger COP-COM distance indicated greater difficulty in controlling body movement when standing as still as possible. In the IW condition, however, participants did not exhibit an increase in backboard angular range or mean angular speed as compared to the B condition, such as those seen in the W condition. Because of this, the W condition seemed to elicit the most extreme changes in balance compared to the B condition. These results also support the idea that adding inertia can mitigate some of the negative effects caused by adding weight [21].

The changes in balance reported here may be explained by mechanical factors and the fact that the postural control system has inherent delays. Adding weight alone moved the average position of the COP anteriorly, indicating a larger average ankle plantar flexor torque and likely larger forces in the plantar flexor muscles. Larger muscle forces are associated with greater force variability [16-18], which is consistent with increases in the range and mean speed of the COP found in the present study. In addition, delays associated with sensory feedback would seem to contribute to greater angular excursion of the backboard prior to a corrective response, which is consistent with the increase in range of backboard angular position found here. Greater angular excursion would also require larger torques to correct [29]. Adding inertia and weight simultaneously exhibited the same general effects on COP-movement as adding weight alone. However, unlike the effect of adding weight alone, the range of backboard angular position and average angular speed of the backboard did not differ from baseline when both inertia and weight were added. This would seem to suggest a beneficial effect of adding inertia by decreasing angular acceleration and therefore angular displacement resulting from delayed sensory feedback. Interestingly, simultaneously adding inertia and weight appeared to exhibit a beneficial effect on backboard motion compared to adding weight alone, yet adding inertia to the baseline condition without adding weight exhibited no apparent beneficial effect. While the reason for this is not immediately apparent, it is possible that further reduction of postural sway in the baseline condition is limited by sensory thresholds and/or muscle force variability rather than any benefit that can be derived from adding inertia. Adding inertia after adding weight, however, did provide some benefit to sway, perhaps because the increased sway induced by adding weight did provide some “room for improvement” with adding inertia. It is also interesting to note that adding inertia alone did not appear to require larger ankle torques to maintain quiet standing because mean COP position and COP range did not increase from the baseline condition.

## Conclusions

Adding inertia and adding weight had different effects on balance. Adding inertia by itself had no measurable effect on center of pressure (COP) movement or backboard movement. Adding weight by itself increased COP movement (indicating greater effort by the postural control system to stand as still as possible) and backboard movement (indicating a poorer ability of the body to stand as still as possible). Adding inertia and weight at the same time increased COP movement, but not backboard movement, compared to the baseline condition. Thus, when adding inertia and weight at the same time, the added inertia appeared to lessen (but did not eliminate) the negative effect of adding weight on balance. These results provide unique insight on how adding mass, and its associated factors of weight and inertia, influence human balance.

## Competing interests

The authors declare that they have no competing interests.

## Authors’ contributions

KEC participated in the design and preliminary testing of the backboard apparatus, data collection, data analysis, data interpretation, and manuscript writing. SLM participated in the design of the backboard apparatus, data collection, and data analysis. MLM participated in the design of the backboard apparatus, data analysis, data interpretation, and manuscript writing. All authors read and approved the final manuscript.
